# Wild boar visits to commercial pig farms in southwest England: implications for disease transmission

**DOI:** 10.1007/s10344-022-01618-2

**Published:** 2022-10-04

**Authors:** Sonny A. Bacigalupo, Linda K. Dixon, Simon Gubbins, Adam J. Kucharski, Julian A. Drewe

**Affiliations:** 1 Royal Veterinary College, University of London, Hatfield, AL9 7TA UK; 2 The Pirbright Institute, Pirbright, Surrey UK; 3 London School of Hygiene & Tropical Medicine, University of London, London, UK

**Keywords:** Camera-trap, Interface, Disease transmission, Pig, Wild boar

## Abstract

Contact between wild animals and farmed livestock may result in disease transmission with huge financial, welfare and ethical consequences. Conflicts between people and wildlife can also arise when species such as wild boar (Sus scrofa) consume crops or dig up pasture. This is a relatively recent problem in England where wild boar populations have become re-established in the last 20 years following a 500-year absence. The aim of this pilot study was to determine if and how often free-living wild boar visited two commercial pig farms near the Forest of Dean in southwest England. We placed 20 motion-sensitive camera traps at potential entry points to, and trails surrounding, the perimeter of two farmyards housing domestic pigs between August 2019 and February 2021, covering a total of 6030 trap nights. Forty wild boar detections were recorded on one farm spread across 27 nights, with a median (range) of 1 (0 to 7) night of wild boar activity per calendar month. Most of these wild boar detections occurred between ten and twenty metres of housed domestic pigs. No wild boar was detected at the other farm. These results confirm wild boar do visit commercial pig farms, and therefore, there is potential for contact and pathogen exchange between wild boar and domestic pigs. The visitation rates derived from this study could be used to parameterise disease transmission models of pathogens common to domestic pigs and wild boars, such as the African swine fever virus, and subsequently to develop mitigation strategies to reduce unwanted contacts.

## Introduction

Contact between wild animals and farmed livestock may result in disease transmission with huge financial, welfare and ethical consequences (Wiethoelter et al. 2015). As an example, the diagnosis and control of bovine tuberculosis in cattle and badgers (*Meles mele*s) currently costs the British taxpayer over £100 million per year (DEFRA 2021). Disease transmission may occur when there is direct or indirect contact between individuals or groups (Craft 2015). A better understanding of the interfaces on and around farms where livestock and wildlife come into contact with each other, and how often these interactions occur, is therefore important (Bacigalupo et al. 2020).

Wild boar (*Sus scrofa*) are one of the most widely distributed mammals globally (Long 2003). Contact between wild boar and livestock is of concern because wild boar can be hosts of many serious pathogens of livestock, including African swine fever virus and classical swine fever virus affecting pigs (Dixon et al. 2019; Postel et al. 2018), *Mycobacterium bovis* (bovine tuberculosis) affecting cattle (Naranjo et al. 2008) and foot-and-mouth disease virus affecting cloven-hoofed livestock (Grubman and Baxt 2004). In addition, wild boars may host zoonotic pathogens (which may be asymptomatic in livestock) such as hepatitis E virus and *Trichinella* (Meng et al. 2009). Wild boar interactions with livestock are also a source of human-wildlife conflict due to crop losses and property damage (Pandey et al. 2016; Thurfjell et al. 2009). Internationally, an increase in the number of conflicts associated with wild boar is expected (Wang et al. 2019), owing to such factors as the opportunistic diet of this species, their rapid population growth and ability to mate with domestic pigs, and their gregarious behaviour which increases the chances of interactions with livestock (Jori et al. 2017).

Knowledge gained from a better understanding of wild boar-livestock contacts has multiple uses. Information of the types of contacts and rates at which they occur can be used to inform mathematical models of multi-host disease transmission (Craft 2015). Monitoring these contacts can be useful in identifying drivers for wild boar contact with livestock (Wyckoff et al. 2009) and risk factors for contacts (Wu et al. 2012), as well as high-risk areas and behaviours that could lead to disease transmission. Quantifying contacts can help target and improve disease prevention strategies, as shown by Barasona et al. (Barasona et al. 2013) when testing the effectiveness of cattle-operated gates at preventing wild boar-cattle interactions at waterholes. These applications could reduce the marked economic loss from diseases potentially transmitted between wild boar and livestock, as well as the public health impact of zoonotic pathogens that may be transmitted between wild boar, livestock and humans (Charrier et al. 2018; Jori et al. 2017). For example, African swine fever outbreaks led to the loss of 12–20% of the global pig herd and a subsequent 10% increase in the food price index of pork in 2018–2019 (Pitts and Whitnall 2019). While contacts between wild boar and livestock have important implications, they are difficult to quantify and characterise because wild boar are elusive and challenging to observe. Understanding interactions between wild boar and livestock is especially important in areas where they have the potential to occur with high frequency, such as where livestock is farmed in close proximity to wild boar habitats and when poor biosecurity measures are implemented. In particular, wild and domestic swine are at risk of inter-population disease transmission because they belong to the same species and share the same community of potential pathogens.

Wild boar have been reintroduced to the Forest of Dean in south-west England in the last 20 years, and the population has increased in size despite ongoing management through culling (Gill and Waeber 2019; Goulding 2003). The wild boar population is a source of human-wildlife conflict as it is an area of high human activity as a tourist destination, as well as being in close proximity to residential areas and farmland (Dutton et al. 2015). This population has also been found to host some pathogens of domestic pigs, such as *Leptospira* Bratislava and pathogens with zoonotic potential, such as Hepatitis E virus (Williamson et al. 2017). There is anecdotal evidence of wild boar activity on farmland, including reports of inter-breeding between wild boar and domestic pigs (Dutton et al. 2015). These farm interactions are concerning due to the potential risk wild boar pose in the transmission of diseases (Croft et al. 2019, 2020; Dutton et al. 2015).

The aim of this pilot study was to determine how often free-living wild boar visited two commercial pig farms in or near the Forest of Dean; the visitation rates derived from this study could be used to parameterise disease transmission models of pathogens common to domestic pigs and wild boar, such as African swine fever virus.

## Materials and methods

### Study area

**Fig. 1:**
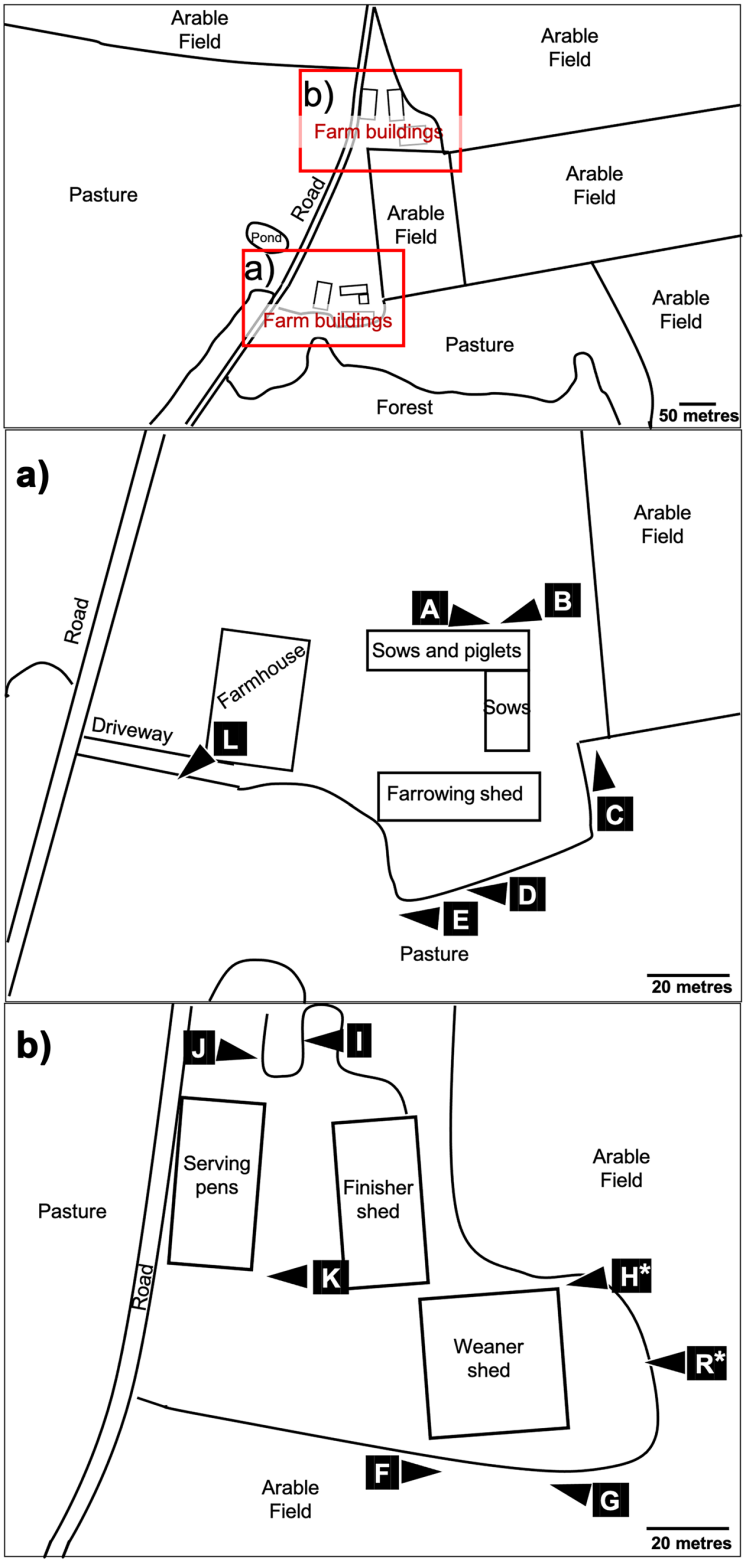
Satellite image of farm 1 and the surrounding area. Insets (**a**) and (**b**) show the locations of camera traps—indicated as white triangles labelled with each camera identification letter (cameras pointed towards the letters). The two camera locations labelled with an asterisk detected 70% of the wild boar activity recorded in this study

**Fig. 2:**
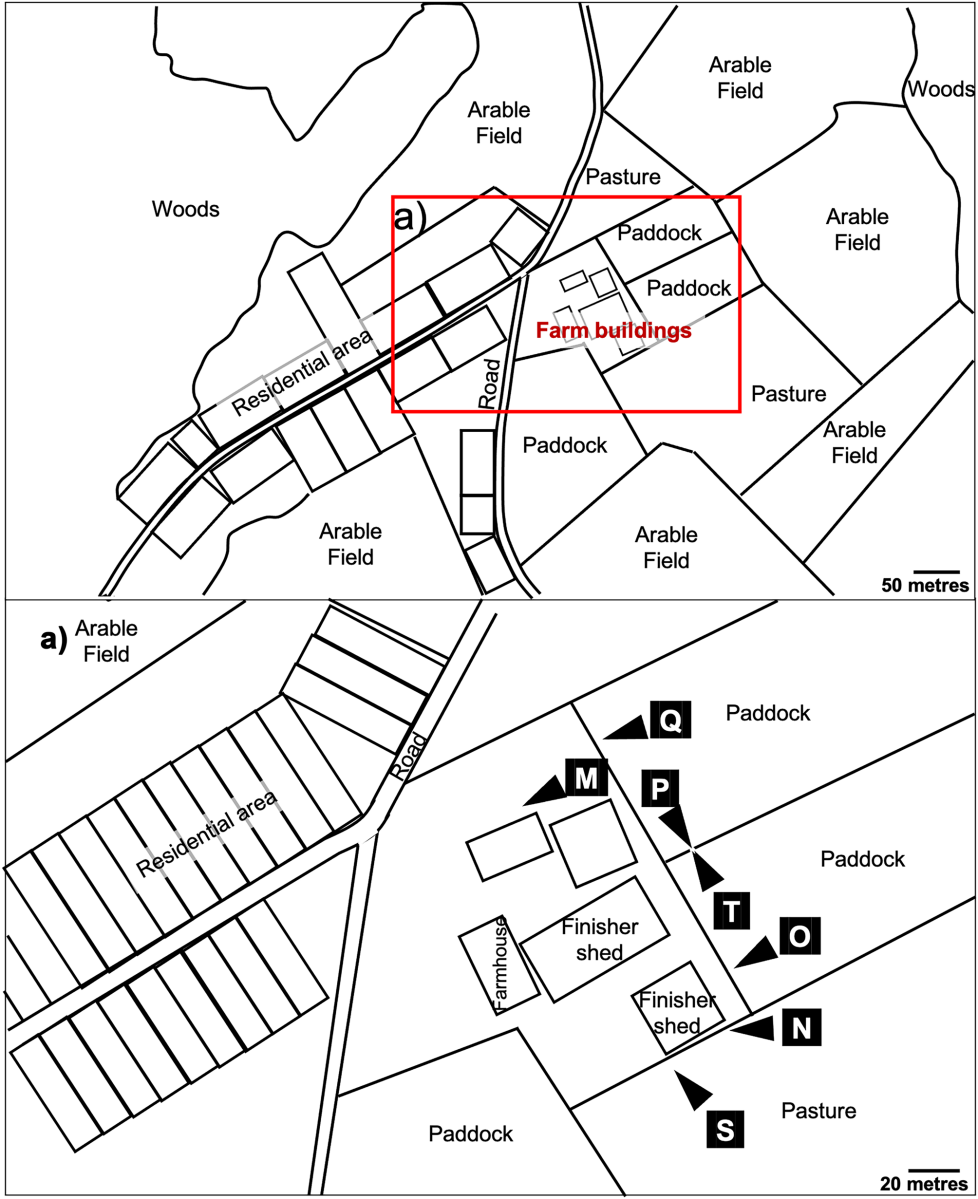
Satellite image of farm 2 and the surrounding area. Inset (**a**) shows the locations of camera traps—indicated as white triangles labelled with each camera identification letter (cameras pointed towards the letters)

This study was conducted on two commercial pig farms, farm 1 and farm 2, that had reported wild boar activity occurring on or around the farm during the previous year. These were the only commercial premises in the area that were not smallholders or backyard premises. Other similar premises were well outside known wild boar ranges. Both sites were within two kilometres of the Forest of Dean in Gloucestershire, UK. The Forest of Dean is a popular tourist destination and hosts the largest population of wild boar in England, estimated at 1172 individuals (95% confidence interval: 885 to 1552) in 2019 (Gill and Waeber 2019). The region is surrounded on three sides by the rivers Severn and Wye, and these, along with a motorway to the north, act as barriers to wild boar expansion. The forest comprises 75km^2^ of mixed broadleaved and coniferous woodland managed by Forestry England. Forestry England carries out population management culls inside the forest throughout the year, and there is anecdotal evidence of shooting of wild boar in surrounding private land, but no private hunting occurs inside the Forest of Dean. Farm 1 contained around 1000 pigs of all ages from piglets to finishers, housed in sheds, and was surrounded on three sides by arable fields and fenced and unfenced pastures used for sheep and cattle grazing (Fig. 1). A road ran adjacent to the farm 1 with a partially fenced pasture beyond. Wild boar were present in the forested area around 50 m from farm 1. Farm 2 contained around 1700 finisher pigs housed in sheds and was located on the edge of a residential village, surrounded on three sides by pasture and paddocks used for grazing sheep and horses, and on one side by residential houses and a road (Fig. 2). Wild boar were known to be present in woodland around 150 m from the farm. Pig sheds at both premises were located on farmyards surrounded by single fencing, and paddocks at farm 2 were also fenced.

### Camera trap survey

The study ran from August 2019 until February 2021. Following a 4-week pilot study involving ten motion-sensing infrared digital cameras (Bolyguard SG520, Boly Inc., SCC, CA, USA) on farm 1, a total of twenty cameras were placed at likely entry points to, and trails surrounding, the perimeter of farmyards on farm 1 (13 cameras: Fig. 1) and farm 2 (7 cameras: Fig. 2). Cameras were placed within 50 m of pigs since farmland within this distance of pigs was used by other livestock, farm workers and their equipment, which could facilitate indirect contact between pigs and wild boar. Each camera location was given a unique letter identification code (from A to T). On farm 1, ten camera traps were deployed in August 2019 (at locations A to J), two in October 2019 (K and L) and one from January 2020 (R). On farm 2, five camera traps were deployed in November 2019 (M, N, O, P and T) and two more in December 2019 (Q and S). Cameras had a field of view of 55 degrees and a detection range of 24 m and were positioned on posts approximately 50 cm above the ground. Cameras were programmed to take three consecutive photographs per trigger event, with a delay of 30 s following a trigger event. Cameras were operational from sunset to sunrise (meaning the time active varied by season). The meteorological definition of season was used, where spring, summer, autumn and winter started on the first day of March, June, September and December, respectively. Camera images were downloaded, and batteries replaced approximately every 8 weeks throughout the study period. Camera trap operation times and malfunctions were recorded in Microsoft Excel (2016).

### Image processing and analysis

Images were organised by camera location ID and camera serial number and were processed and analysed using R (version 4.0.0) (R Core Team 2020). Images were copied and renamed based on location ID and image creation date using the imageRename function of the camtrapR package (version 2.0.3.06) (Niedballa et al. 2016). Images were manually inspected for wild boar by one person (SAB), and details of the images containing wild boar were tabulated, metadata extracted and data explored and visualised using camtrapR. Images containing at least one wild boar, captured within 1 min of each other by the same camera, were considered as one detection (1 min was the smallest non-zero value allowable in camtrapR) since it was not possible to distinguish between individual wild boar or determine the size of the group present from the still images and to account for multiple photographs per trigger event. For each camera location, the number of operational camera nights and the number of wild boar detections were recorded and the results were visualised using the ggplot2 (version 3.3.2) and tidyverse (version 1.3.0) packages (Wickham 2016; Wickham et al. 2019). For each farm, detection rates for each camera location, month and season were calculated by dividing the number of detections by the number of operational camera-nights; differences between rates were identified by inspecting for non-overlapping ranges of 95% confidence intervals. A wild boar visit was defined as any night where at least one wild boar was detected on a farm, since the same wild boar could have been detected multiple times at multiple cameras in the same night. Monthly visit rates were calculated by dividing the number of visits with the number of days where at least one camera was operational. Monthly indirect contact rates between wild boar and domestic pigs were assumed to be the same as the monthly visit rate; therefore, an indirect contact was defined as wild boar being less than 50 m away from pigs. Direct nose-to-nose contacts between wild boar and pigs were not detectable in this study due to the placement of cameras. Where reported, 95% confidence intervals for detection rates and visit rates were calculated using the Poisson test.

## Results

### Overview of camera trap operations

**Fig. 3:**
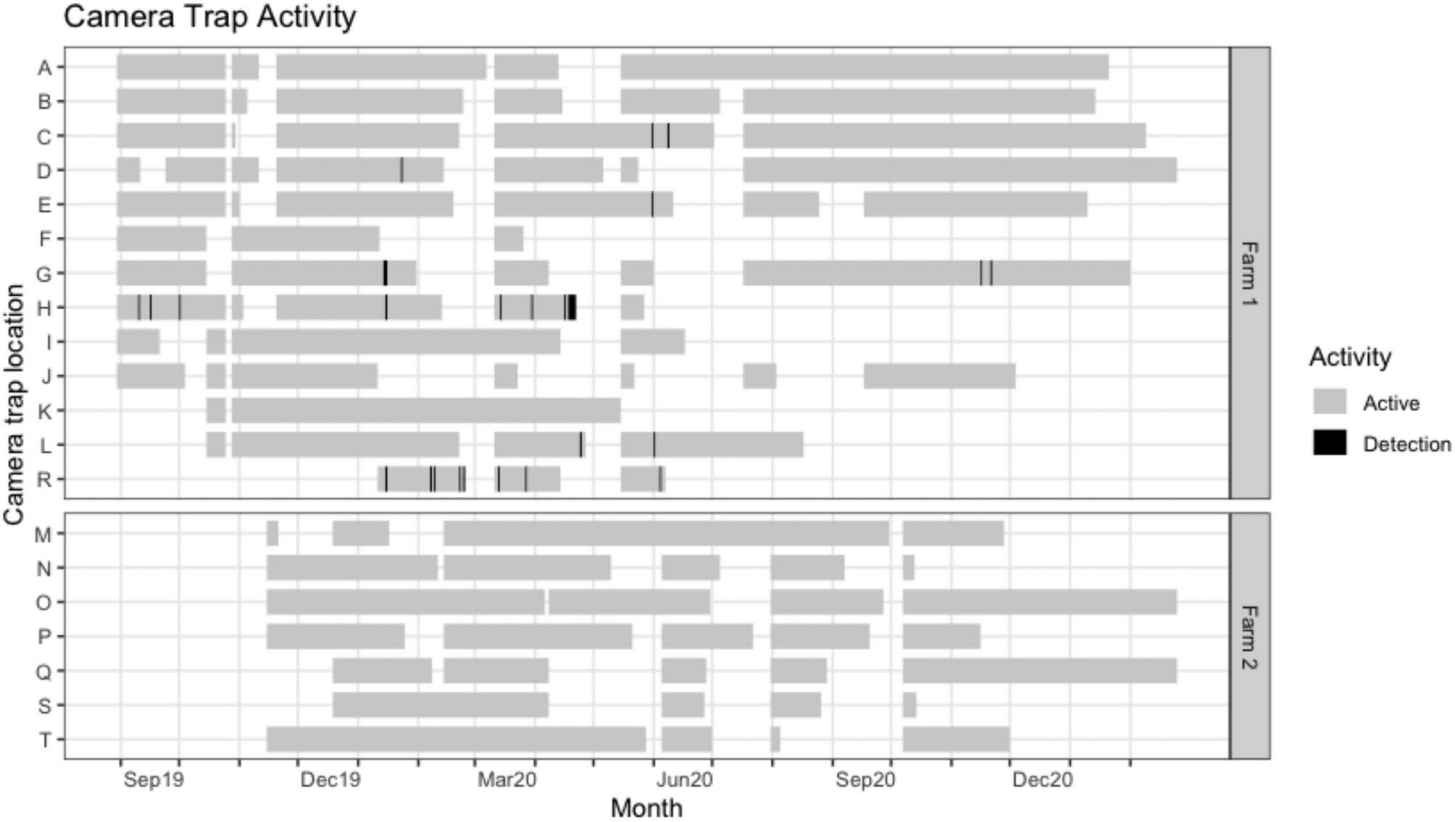
Periods of operation (grey bars) for the 20 camera traps with the timing of wild boar detections (black lines) on farm 1 and farm 2 over the study period from September 2019 to February 2021. No wild boar were detected by the 7 cameras on Farm 2

Camera operation durations and timings of wild boar detections are shown in Fig. 3. Six cameras on farm 1 failed to capture images after March 2020 (F), May 2020 (H and K), June 2020 (I and R) and August 2020 (L). The remaining cameras remained operational until between October 2020 and February 2021.

During the study period, cameras were active for 3990 camera nights on farm 1 and 2040 camera nights on farm 2. Cameras were triggered over 130,000 times, resulting in over 400,000 images, of which 40 (approximately 0.03% of tigger events) were wild boar. Monthly camera trap effort ranged from 137 to 372 camera nights.

### Detection of wild boar activity on farms

**Fig. 4:**
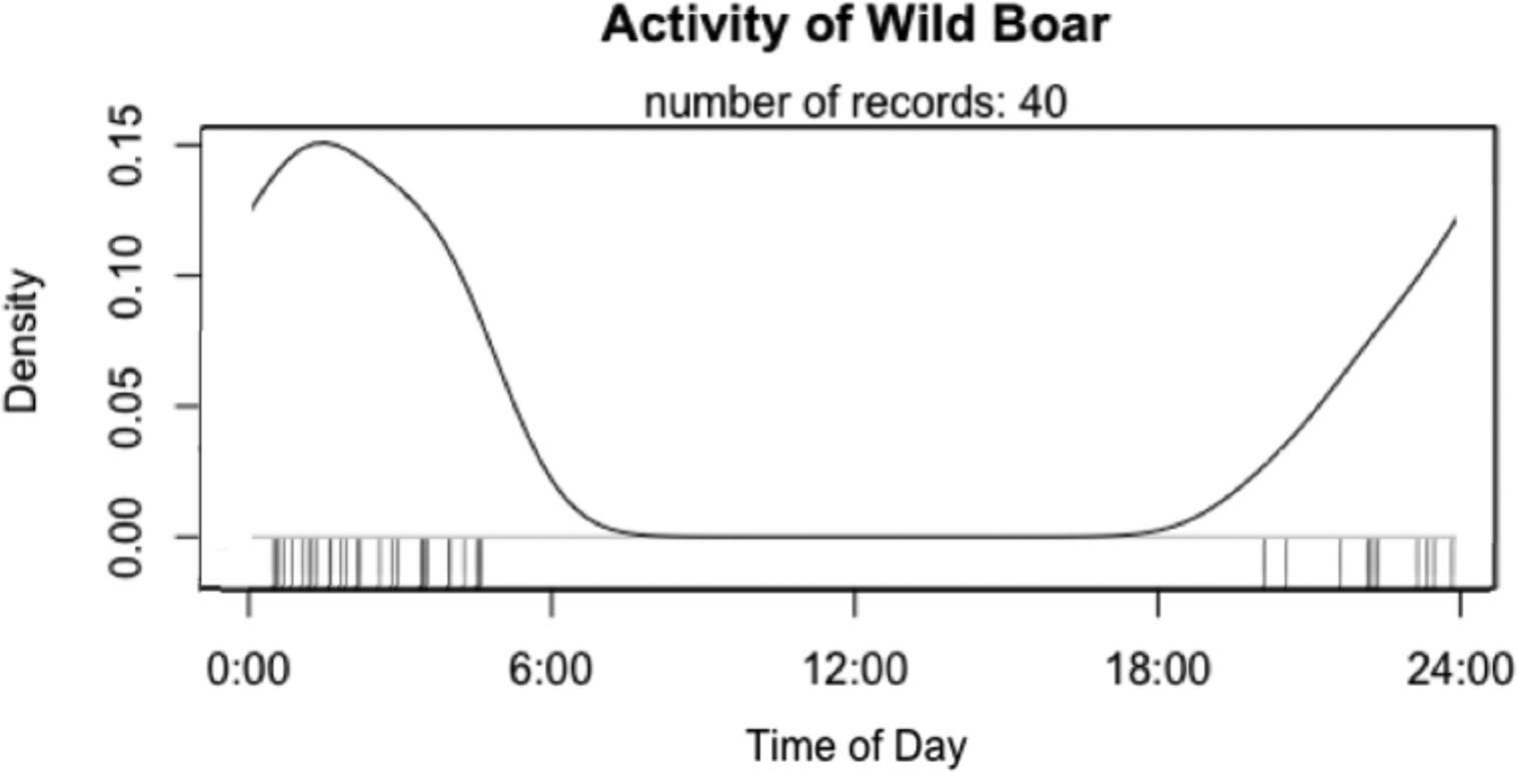
Daily pattern (relative frequency by time) of wild boar activity on farm 1. The vertical ticks along the x-axis indicate the timing of wild boar detections captured by the cameras. Density along the y axis is calculated as the number of detections within a time frame as a proportion of the total number of detections

There were 40 wild boar detections on farm 1, spread across 27 nights (Table 1). There was one detection per night recorded on 20 of these nights. On seven nights, wild boar were detected multiple times (from 2 to 7 times) during the same night, either by the same camera (for example, three visits were recorded at location R on 22 February 2020) or by multiple cameras (for example, three cameras recorded a total of 7 visits on 15 January 2020). Three images contained two wild boar in the same image, while the remainder contained a single wild boar per image (Table 1). All of the 40 wild boar detections occurred during hours of darkness between 20:04 and 04:34, with a peak of activity (8 out of 40 detections) occurring between 01:00 and 02:00 (Fig. 4). No wild boar activity was detected on farm 2.

**Table 1:** Chronology of wild boar visits recorded by camera traps on farm 1, including the number of days since the last detection and the maximum number of wild boar observed in the images

Date	Camera location	Time (24-h clock)	Number of wild boar in image	Days since the last detection
10/09/2019	H	01:01	2	-
16/09/2019	H	23:19	1	6
01/10/2019	H	22:20	1	15
14/01/2020	G	01:47	1	105
15/01/2020	G H R	04:34 00:29, 03:24, 04:30 00:28, 03:23, 04:30	2	1
23/01/2020	D	03:26	1	8
07/02/2020	R	01:34, 01:36	2	15
09/02/2020	R	01:54	1	2
22/02/2020	R	22:07, 22:11, 22:15	1	13
24/02/2020	R	00:40	1	2
13/03/2020	R	23:48	1	17
14/03/2020	H	00:49	1	1
27/03/2020	R	03:31	1	13
30/03/2020	H	23:26	1	3
16/04/2020	H	02:34	1	17
18/04/2020	H	20:31	1	2
19/04/2020	H	20:04	1	1
20/04/2020	H	03:55, 03:57	1	1
21/04/2020	H	04:14	1	1
22/04/2020	H	01:11	1	1
24/04/2020	L	21:34	1	2
31/05/2020	C E	01:10 01:17	1	37
01/06/2020	L	02:48, 02:55	1	1
04/06/2020	R	23:53	1	3
08/06/2020	C	23:07	1	4
16/11/2020	G	00:32	1	159
21/11/2020	G	02:06, 02:11	1	5

**Fig. 5:**
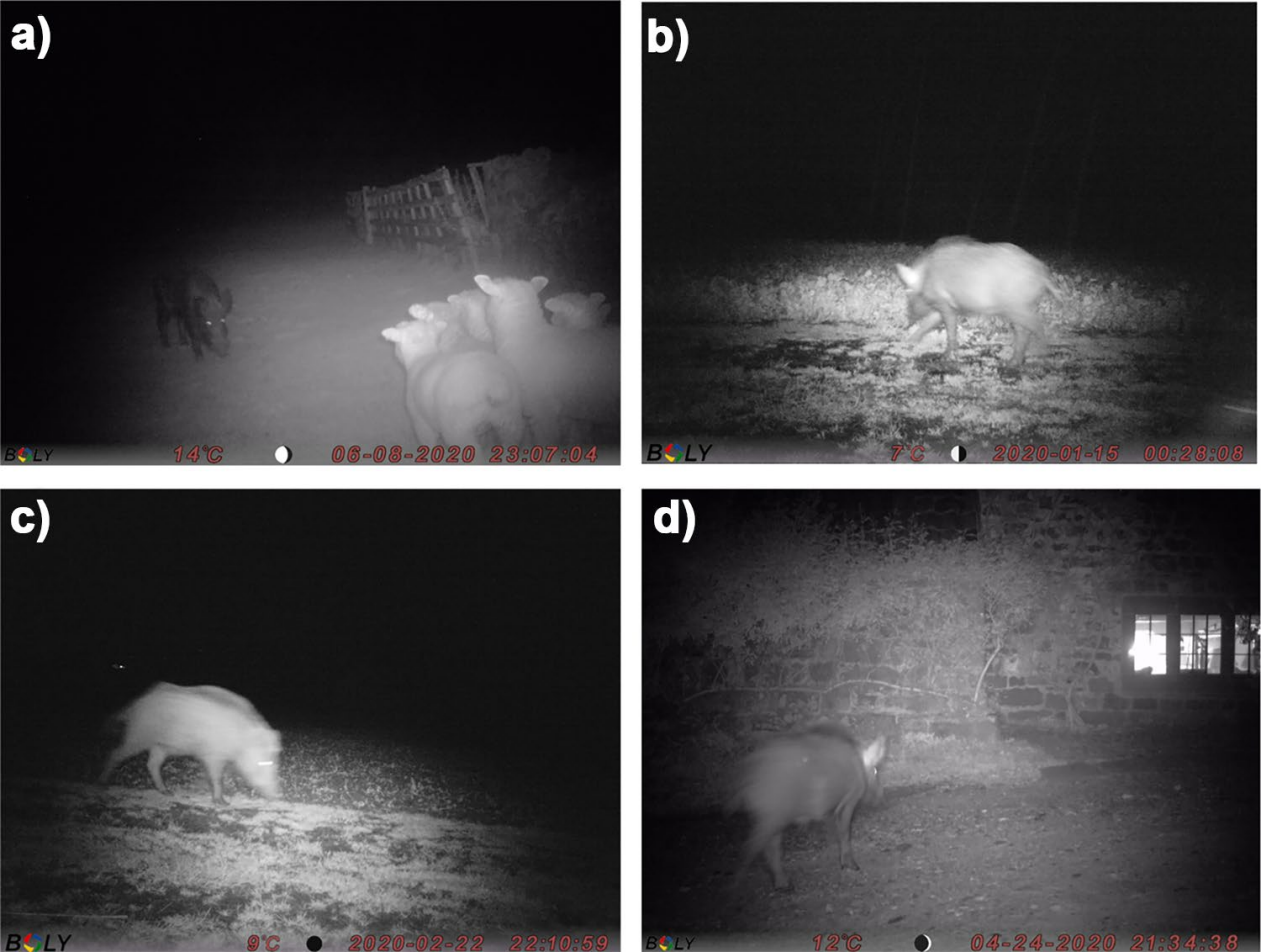
Wild boar images from farm 1 showing **a** wild boar [left of photo] facing a cluster of sheep next to a farmyard gate (location C); **b** and **c** wild boar walking by a crop field near the weaner shed (location R); **d** wild boar on the driveway approaching some farm buildings (location L)

On farm 1, wild boar activity was detected by cameras at one or both locations H and R on the edge of the same crop field on 19 of the 27 nights (70%) that activity was detected at the farm (Table 1). These cameras were positioned within ten to twenty metres of housed domestic pigs. The camera at location G (in the crop field adjacent to locations H and R) detected five instances of wild boar activity over four nights. Wild boar were detected by the camera at location L on the farm driveway next to the farmhouse three times over two nights, and the remaining four detections took place in a field between the farm and the forest by cameras at locations C, D and E. Locations A, B and K were on the farm itself, rather than at the periphery, and cameras here did not detect any wild boar activity. Despite the proximity of cameras to pigs, direct nose-to-nose contacts between pigs and wild boar were not detectable or observed. Most images showed wild boar walking past the farm, although one image showed a wild boar interacting with sheep, and the camera at location L captured a wild boar that appeared to be moving onto the farm rather than walking past it (Fig. 5).

**Fig. 6:**
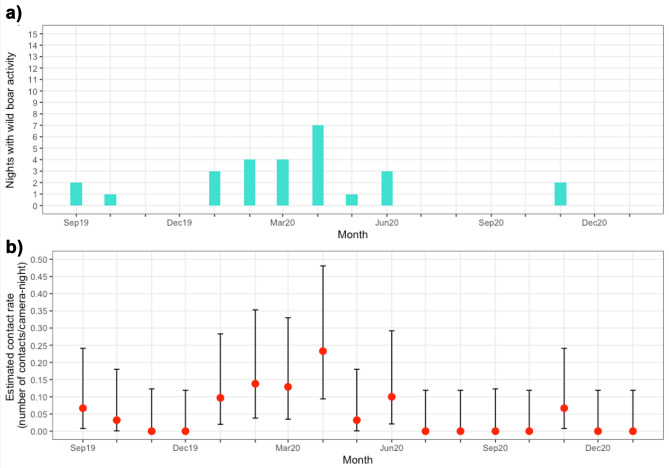
**a** Number of nights with wild boar activity on farm 1 per month. **b** Estimated visit/contact rates for farm 1 per month with 95% confidence intervals calculated with the Poisson test. August 2019 and February 2021 are omitted from both these figures because there were very limited data in these part-months (only 20 and 33 camera trap nights, respectively)

The number of nights where wild boar activity was detected ranged from zero to seven per month, with a median of one night of wild boar activity per month (Fig. 6). April 2020 had seven nights of wild boar activity and a detection rate of 0.031 (95% C.I.: 0.012, 0.064) detections per camera-night or 32 (95% C.I.: 16, 80) camera-nights between detections. There was no wild boar activity detected from October 2019 to January 2020 (105 days), July to October 2020 (159 days) or from December 2020 to February 2021 (105 days) (Table 1). The nightly visit rates by month ranged from 0.032 (95% C.I.: 0.001, 0.180) to 0.226 (95% C.I.: 0.091, 0.465) (Fig. 6). No difference in the number of wild boar visits per month to farm 1 was detected, nor were any seasonal differences in wild boar detections observed (Table 2). Spring 2020 had the most nights of wild boar activity with 12 of the 27 nights (44%), and an estimated detection rate of 0.020 (95% C.I.: 0.011, 0.028), or 51 (95% C.I.: 30, 92) camera-nights between detections. Aside from winter 2020, where no activity was detected, autumn had the lowest detection rates of wild boar with 0.004 (95% C.I.: 0.001, 0.011) detections per camera-night in 2019 and 0.005 (95% C.I.: 0.001, 0.014) in 2020. The highest estimated rate of wild boar visits occurred at locations R and H, where there were 0.127 (95% C.I.: 0.068, 0.218) and 0.075 (95% C.I.: 0.042, 0.123) detections per camera-night, respectively (Fig. 1 and Table 3). Estimated detection rates at other locations where activity was detected ranged from 0.002 (95% C.I.: 0.0001, 0.013) to 0.013 (95% C.I.: 0.004, 0.030) (Table 3).

**Table 2:** Seasonal variation in detection rates on farm 1 displayed as detections per night and nights between detections. A maximum of one detection per night was used in these calculations

Season	Camera-nights	Nights of wild boar activity	Estimated Detection Rate—detections per camera-night (95% confidence Interval)	Camera- nights between detections (95% confidence interval)
Autumn 2019	807	3	0.004 (0.001, 0.011)	269 (92, 1304)
Winter 2019	941	7	0.017 (0.010, 0.028)	58 (36, 102)
Spring 2020	721	12	0.020 (0.011, 0.033)	51 (30, 92)
Summer 2020	506	3	0.008 (0.002, 0.020)	126 (49, 461)
Autumn 2020	605	2	0.005 (0.001, 0.014)	201 (69, 974)
Winter 2020	360	0	0.000 (0.000, 0.010)	0.00 (98, NA)

**Table 3:** Camera trap activity and rates of wild boar visits detected on the two study farms

Study Site	Location	Total nights deployed	Nights active (%)	Number of wild boar detections	Estimated detection rate—detections per camera- night (95% confidence interval)	Number of camera-nights between detections (95% confidence interval)
Farm 1	A	510	462 (91)	0	0 (0.000, 0.007)	0 (125, NA)
	B	503	427 (85)	0	0 (0.000, 0.009)	0 (116, NA)
	C	529	472 (89)	2	0.004 (0.001, 0.015)	236 (65, 1949)
	D	545	431 (79)	1	0.002 (0.000, 0.013	431 (77, 17,024)
	E	499	397 (80)	1	0.003 (0.000, 0.014)	397 (71, 15,681)
	F	209	137 (66)	0	0 (0.000, 0.027)	0 (37, NA)
	G	521	385 (74)	5	0.013 (0.004, 0.030)	77 (33, 237)
	H	271	201 (74)	15	0.075 (0.042, 0.123)	13 (8, 24)
	I	292	234 (80)	0	0 (0.000, 0.016)	0 (63, NA)
	J	462	234 (51)	0	0 (0.000, 0.016)	0 (63, NA)
	K	213	210 (99)	0	0 (0.000, 0.018)	0 (57, NA)
	L	307	268 (87)	3	0.011 (0.002, 0.033)	89 (31, 433)
	R	148	102 (69)	13	0.127 (0.068, 0.218)	8 (5, 15)
Farm 2	M	379	316 (83)	0	0 (0.000, 0.012)	0 (86, NA)
	N	333	248 (75)	0	0 (0.000, 0.015)	0 (67, NA)
	O	468	425 (91)	0	0 (0.000, 0.009)	0 (115, NA)
	P	367	306 (83)	0	0 (0.000, 0.012)	0 (83, NA)
	Q	434	298 (69)	0	0 (0.000, 0.012)	0 (81, NA)
	S	300	166 (55)	0	0 (0.000, 0.022)	0 (45, NA)
	T	382	281 (74)	0	0 (0.000, 0.013)	0 (76, NA)

Assuming that all wild boar visits were detected, the estimated number of nights where wild boar visited farm 1, or the indirect contact rate, ranged from 0 (95% C.I.: 0, 4) to 7 (95% C.I.: 3, 14) contacts per month with a mean of 1.6 and a median of 1 contact per month.

In comparison to wild boar detections, other mammals were detected at considerably higher rates. Domestic livestock were the most frequently detected species on both farms. On farm 1 and farm 2, rabbits (*Oryctolagus cuniculus*) and foxes (*Vulpes vulpes*) were detected multiple times per night nearly every night. Badgers were detected multiple times a night nearly every night at farm 1 and sporadically on farm 2. Fallow deer (*Dama dama*) were detected on farm 1 on substantially more nights than wild boar, often multiple times a night, but were not detected on farm 2. One polecat (*Mustela putorius*) was detected on farm 2, and unidentifiable rodent species were occasionally detected on both farms.

## Discussion

### Use of cameras to monitor wild boar

Using motion-detection cameras, we quantified wild boar activity over 18 months on two commercial pig farms in southwest England and detected wild boar on only one of these farms. A previous study using camera traps to measure wild boar abundance in the Forest of Dean and other UK woodlands suggested nine cameras per square kilometre were necessary to establish population density (Massei et al. 2018). The density of camera traps in this study was approximately 450 per square kilometre on farm 1 and 300 per square kilometre on farm 2, so we can have some confidence that wild boar would be detected if they were on or near the farms.

### The potential for contacts to infer disease transmission rates

Establishing the frequency with which contacts (whether direct or indirect) between wildlife and livestock occur can be used to inform mathematical models of pathogen transmission where the disease affects multiple species, as used in bovine tuberculosis transmission models utilising contact rates between cattle and badgers (Craft 2015; Wilber et al. 2019). While some studies have used disease outbreak data to assess the likelihood of transmission events (for example Vergne et al. 2017), few studies have used transmission rates between wild boar and livestock in disease transmission models, which is a limitation in models where wild boar are considered to play an important role such as in African swine fever transmission (Hayes et al. 2021). One exception is a study by Taylor et al. (Taylor et al. 2021) which used contact rates from wild boar and pigs to infer a wild boar-to-pig transmission rate for African swine fever in Poland. This data, while useful for the model, was derived from a study of extensively farmed pigs in a savanna-type habitat (Kukielka et al. 2013), and more contact data from a variety of systems and farm types is needed. In our study, wild boar were recorded on farm 1 up to seven times per month (median of 1) and this may be an underestimate; up to 14 visits per month could be considered reasonable based on 95% confidence intervals of the estimated visit rate (Fig. 6), and visits by multiple wild boar per night were not considered due to the difficulty of identifying individual wild boar. These figures suggest a median visit rate between wild boar and domestic pigs of 1 visit per 30 days. In our study, most wild boar activity occurred between ten and twenty metres of housed domestic pigs where there is potential for indirect contact and pathogen transmission. On farm 1, soiled bedding from pig pens was kept outside the farmyard perimeter, which would be the most likely access to fomites or uneaten feed by wild boar. Nose-to-nose contact through fencing was possible on farm 1 (though not detectable in this study) if wild boar gained access to the farmyard. Not all visits or contact events necessarily lead to an infection transmission event, and some activities and types of contact may be more likely to lead to disease transmission than others (Craft 2015). For example, while it may be possible for pathogen transmission to occur from wild boar observed in our study via aerosol, or from infected fomites and excreta left on farmland to be brought to pig sheds by people or machinery, the transmission may be more likely if the wild boar were seen to be in direct contact with pigs or shared feed or water. Nevertheless, the visit rates observed in this study could be considered as the upper bound for the average indirect contact rate (with a minimum of 0 per 30 days and a maximum of 7 per 30 days). The probability of disease transmission for each contact event would need to be observed or calculated to find the actual transmission rate and would depend on the pathogen and transmission route. Most of the 27 nights of wild boar activity recorded in the present study were preceded by fewer than 17 nights of inactivity, with only three nights where there were more than 17 nights between detections. If there was a delay of more than 17 days between the introduction and detection of an exotic pathogen to a farm (for example the median time for detection of African swine fever on pig farms is 13 days, with a likely maximum of 23 days (EFSA 2021), a risk of disease transmission from domestic pigs to wild boar would exist before the disease is detected on the farm.

### Identifying risk factors for wild boar activity near pig farms

Wild boar are opportunistic and risk factors for activity near pig farms include foraging for water and food, including crops near enclosures, and for breeding purposes (Wu et al. 2012; Jori et al. 2017). While this pilot study did not explicitly aim to identify risk factors, our findings go some way to identifying possible risk factors for wild boar activity on farms, and these could be usefully quantified in follow-up studies. Camera trap studies involving wild boar interactions with livestock in different contexts in Europe have used the data to identify areas and times of increased visitation rates, for example, monitoring visits to cattle farms to investigate bovine tuberculosis transmission (Kukielka et al. 2013; Payne et al. 2016) or wild boar interactions with extensively farmed pigs (Cadenas-Fernandez et al. 2019). In our study, there appeared to be increased activity in areas of farm 1 adjacent to crop fields, and in most cases, the wild boar were walking along tracks past the farm rather than spending extended periods of time on the farm, which is similar to findings from a study near outdoor pig farms in Switzerland by Wu et al. (2012). This information may be useful in targeting strategies to prevent wild boar and domestic pig contacts, whether for purposes of mitigating disease transmission or farm damage as demonstrated by Carrasco-Garcia et al. (2016) who examined extensively farmed livestock in Mediterranean conditions. Tracks where wild boar were detected were also used by humans and farm vehicles, and these could have a role in indirect transmission of infectious wild boar urine or faeces onto the farm. There was very little wild boar activity detected on the farm compared to the farm perimeter, so potentially, more risky transmission events such as direct contact or shared use of feed and water may occur infrequently. These types of contact were not explicitly monitored or detectable in this study: water points have been found to be focal areas for indirect contacts between wild boar and livestock on pastures in Spain and France (Kukielka et al. 2013, Payne et al. 2016) but not in others (Varela-Castro et al. 2021). Identifying risk factors and potential reasons for the difference in wild boar activity between farm 1 and farm 2 would be useful but is not evident from this study. The lack of wild boar detections on farm 2, despite recent and continued wild boar sightings and crop damage in the surrounding area, could be due to the proximity of the residential area, the absence of crops in the immediate area of the farmland, or the presence of secondary fencing surrounding livestock pastures. Farm 2 was a finishing farm with no breeding sows. If breeding sows are responsible for attracting wild boar visits to pig farms (Jori et al. 2017), this might explain the lack of wild boar detections on farm 2.

As far as we are aware, this is the first study to use camera traps to document wild boar activity on domestic pig farms in Britain. The data collected by this study will be useful to inform the development of disease transmission models that involve contact between wild boar and domestic pigs, for example through the incorporation of minimum and maximum visitation rates of wild boar to pig farms.

### Limitations

A limitation of this study was the small number of study sites and low numbers of wild boar visits detected, particularly at farm 2, which means conclusions made may not be representative of what happens at other farms in the area. There are very few (if any) other pig farms of this size in the area, so the selection of study sites was limited. Our study focused on detecting wild boar near housed domestic pigs, and at farm entry and exit points, so it was necessary to position cameras in areas of high human and livestock activity. This resulted in many irrelevant trigger events and reduced the battery life of cameras which resulted in several cameras not recording continuously throughout the study period, leading to an imbalance in camera effort between months and seasons (Fig. 3). Camera trap failures have occurred in other wild boar and domestic pig contact studies (Engeman et al. 2011), and data may also be missed if the camera trigger speed is slow or interval between triggers is long, so some wild boar activity may have been missed. For these reasons, it is difficult to assess study power, although confidence is increased due to the camera trap effort used, the high density of cameras across the study area and the relatively high rates of visitations detected of other wildlife species such as rabbits, badgers, foxes and fallow deer on both study farms. In order to increase the chances of detecting wild boar, many camera traps in this study were placed over a relatively small area which nullifies the assumption of independence (Sollmann 2018). This meant that it was likely that multiple cameras detected the same wild boar as they moved around the farm, which is supported by the close timings of detections at locations G, H and D. This lack of independence between cameras within each farm and the inability to distinguish between individual wild boar, along with the small sample size, mean that it was not possible to establish whether visits were made by the same individual or group of wild boar or to quantifiably identify risk factors, and so the study remained descriptive.

### Areas for further study

Wild boar activity signs were reported by workers on both farm 1 and farm 2, but these were outside the range of the cameras. While the methods used in this study have been useful to collect information on nearby contacts and visits to domestic pig facilities, monitoring these areas with cameras or other observational methods may provide more information since other methods have been shown in certain circumstances to be more sensitive than camera-traps (Payne et al. 2018). Global positioning system collars have been used successfully in wild boar and could be used in conjunction with camera traps to monitor wild boar locations continuously, so cameras can be used to verify activity at specific areas of high disease transmission risk. This would be similar to methods used in Texas where spatial data was collected using GPS-collared feral swine while cameras monitored interactions of feral swine with pig pens (Wyckoff et al. 2009). It would also be useful to collect more detailed data on wild boar behaviours around or on farm which could be achieved using video surveillance rather than still images, particularly if wild boar are suspected to be spending significant portions of time in one place (Erdtmann and Keuling 2020). In order to get a broader picture of wild boar behaviour around domestic pigs, studying other farm types where pathogen transmission may be more likely to occur, such as outdoor piggeries or small holders should be considered.

### Conclusion

The results of this camera-trap study on pig farms near the Forest of Dean confirm wild boar visit commercial pig farms, and therefore, there is potential for contact and pathogen exchange between wild boar and domestic pigs. The visitation rates derived from this study could be used, along with disease prevalence data from wild boar and domestic pigs, to parameterise disease transmission models of pathogens common to domestic pigs and wild boar, such as the African swine fever virus, and subsequently to develop mitigation strategies to reduce unwanted contacts.

## Data Availability

Not applicable.
